# Role of Purinome, A Complex Signaling System, In Glioblastoma Aggressiveness

**DOI:** 10.3389/fphar.2021.632622

**Published:** 2021-02-05

**Authors:** Patricia Giuliani, Marzia Carluccio, Renata Ciccarelli

**Affiliations:** ^1^ Department of Medical, Oral and Biotechnological Sciences, ‘G. D’Annunzio’ University of Chieti-Pescara, Chieti, Italy; ^2^ Center for Advanced Studies and Technology (CAST), ‘G. D’Annunzio’ University of Chieti-Pescara, Chieti, Italy

**Keywords:** glioblastoma multiforme (GBM), purinome, adenine-based compounds, purine metabolizing enzymes, purinergic receptors

## Introduction

Many recent papers dealing with glioblastoma multiforme (GBM), the most common/lethal human brain tumor, are aimed at identifying new druggable targets, hopefully useful for more effective therapies than the current ones. In this context, the purinergic system, present in all living cells, is arousing considerable interest, mainly in relation to adenine-based compounds. It is composed of:

1. intracellular purine nucleotides, nucleosides and nucleobases involved in fundamental biological processes such as cell duplication, chemical energy supply, intracellular signaling, and protein metabolism regulation (Frenguelli and Dale, 2020);
2. extracellular purines (the same found intracellularly), among which adenine-based nucleotides/nucleosides behave as signal molecules interacting with specific receptors, namely P1 and P2 for adenosine (ADO) and ATP/ADP, respectively (Burnstock, 2018);
3. a wide array of intra/extracellular metabolizing enzymes (Yegutkin, 2014);
4. transporters, mostly deputed to regain nucleosides/bases to restore the intracellular purine pool (Choi and Berdis, 2012).

The advent of the “omic” era has induced scientists to indicate this complex network as “purinome”, to emphasize that its components act in close/interactive fashion. This concerted activity maintains cells in homeostatic/physiological conditions, while purinome imbalance causes/is implicated in profound alterations of normal cell biological activities, thus contributing to malignant cell transformation toward more aggressive phenotypes, as shown in different tumors (Boison and Yegutkin, 2019; Campos-Contreras et al., 2020), including GBM (Giuliani et al., 2018; Zhou et al., 2020). Here, we exemplified some emerging alterations of purinome, highlighting how they influence GBM progression, in order to outline a picture, which can be the natural premise for suggesting new checkpoints of GBM aggressiveness.

## Role of Adenosine TriPhosphate Metabolizing Ecto-Enzymes and Signals in Glioblastoma Aggressiveness

Notoriously, nucleotides, once released from cells, act on different P2 receptors divided into two sub-families including eight metabotropic P2Y and seven ionotopic P2X receptors (Burnstock, 2018). Nucleotide receptor activity is finely tuned by ectoenzymes (Zimmermann et al., 2012). Of these, ectonucleoside triphosphate diphosphohydrolase (E-NTPDases) belong to the CD39 family that comprises eight subtypes, of which NTPDases1, 2 and 3 are present in the brain. Moreover, three subtypes of ectonucleoside pyrophosphatase/phosphodiesterases (E-NPPases) have been identified, being NPP2 and 3 expressed in brain glial cells (Braganhol et al., 2020), whereas tissue-nonspecific alkaline phosphatase (NTAP) is abundant in brain and prevailingly involved in the regulation of neuronal activity (Fonta et al., 2004). Finally, ecto-5ʹ-nucleotidase/CD73 (ecto-5ʹ-NT/CD73) is also a selective mesenchymal marker that, in tandem with CD39, generates ADO, thus contributing to the immunosuppressive potential of mesenchymal stromal cells (Kakiuchi et al., 2020).

**FIGURE 1:**
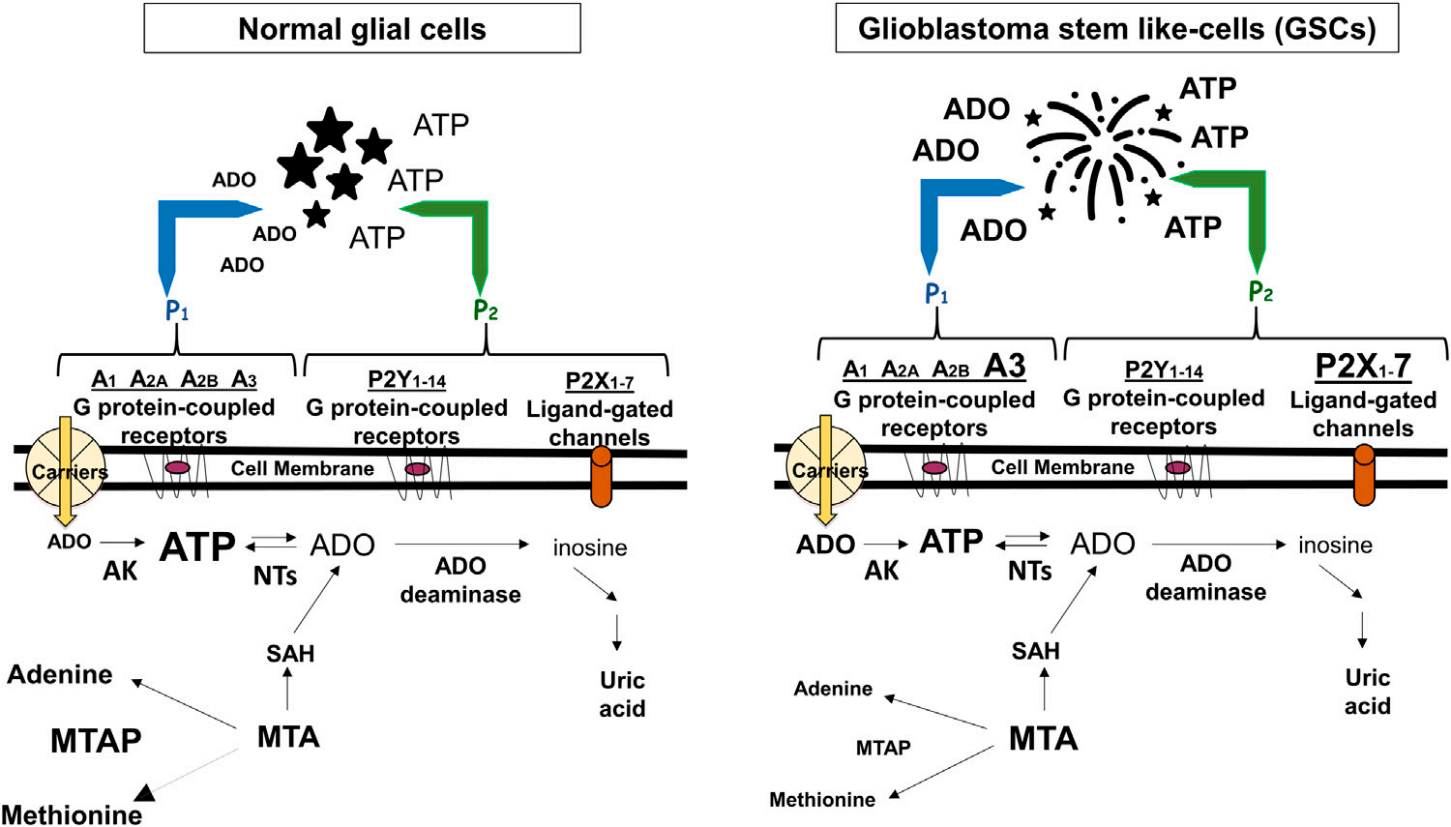
Adenine-based purines are ubiquitous compounds present at intra- and intracellular levels in normal (i.e., astrocytes) and tumoral (i.e., GSCs) cells. Left panel: ATP is released by virtually all cell types, and is usually metabolized by ecto-enzymes into adenosine (ADO) that is regained by cells through selective transporters (carriers). Right panel: In tumors, including GBM, extracellular purine amount is increased in pericellular tumor fluid by cell membrane leakage and also by low metabolism of ATP by ecto-nucleotidases coupled to increased activity of the ectoenzyme (CD73) converting AMP into ADO. The interaction of extracellular ATP or ADO with specific receptors, the expression of which has been found increased in GSCs such as ADO/A3 and/or ATP/P2X7 receptors, activates processes contributing to transform tumor cells in phenotypes more aggressive and resistant to chemotherapy. Additionally, GBMs frequently show homozygous deletion of methylthioadenosine phosphorylase (MTAP), an enzyme working in the purine/methionine salvage pathway to convert methylthioadenosine (MTA) formed during polyamine biosynthesis into adenine and methionine. MTAP loss favors GSC formation with increased aggressiveness (Hansen et al., 2019) and is associated with poor clinical outcome of GBM patients (Zeppernick et al., 2008). In both panels, changes in the receptors expression or purine levels are highlighted by increasing or decreasing the character size and/or by using bold characters. AK, adenosine kinase; NTs, nucleotidases; SAH, S-adenosyl hydroxylase, converting MTA into adenosine.

Alterations in these extracellular/membrane purinome components are evident in GBM. While in normal cerebral tissue extracellular ATP levels are determined by nucleotide release from healthy neural cells (Burnstock, 2020), in pathological conditions including tumors, ATP levels are increased also through membrane leakage of damaged/dying cells, mainly in the hypoxic tumor core (Di Virgilio et al., 2018). However, differently from normal cultured astrocytes (Morrone et al., 2006), a low extracellular ATP metabolism was found in glioma cells (Wink et al., 2003). Further findings confirmed that such metabolic reduction is involved in GBM growth and progression (Braganhol et al., 2020) (Figure 1). Again, GBM cell lines and stem-like cells (GSCs, which support tumor recurrence) (Taga and Tabu, 2020), are sensitive to cytotoxicity of high ATP concentrations, while lower nucleotide levels (below 1 mM) would favor tumor growth (Morrone et al., 2005). Accordingly, co-injection of apyrase, an ATP/ADP scavenger, in C6 rat glioma experimental model, reduced tumor size when compared with that from rats untreated or exposed to inactivated apyrase (Morrone et al., 2006).

In parallel, other findings demonstrated that ATP receptors, i.e., the subtypes P2Y1, P2Y2, P2Y12 and P2X7, are important in GBM pathology (Giuliani et al., 2018; Braganhol et al., 2020). Among them, the P2X7 receptor (P2X7R) subtype, coupled to a pore the opening of which allows entry of molecules up to 900 Da inside cells, is assuming particular relevance in GBM and other tumors (Di Virgilio, 2020). P2X7R activation in glioma cells mediates multiple effects, ranging from cell death to survival and immune system modulation (D’Alimonte et al., 2015; Bergamin et al., 2019). Noteworthy, difference in the responses triggered by P2X7R highly depends on its expression levels (Andrejew et al., 2020). Accordingly, GSCs from primary GBMs show enhanced P2X7R expression (D’Alimonte et al., 2015) and in cells exposed to the P2X7R agonist, 2' (3')-O-(4-benzoylbenzoyl)-ATP (BzATP), the expression of markers associated to epithelial-to-mesenchymal transition (EMT), a process contributing to GSC malignancy, was up-regulated as well as that of subunits of two main human P2X7R splice variants, P2X7A and P2X7B (Ziberi et al., 2019). This condition might favor A/B subunit assembly into a heterotrimeric P2X7R with major sensitivity toward agonists and cell energy support. All aforementioned findings suggest a crucial P2X7R role in GBM recurrence/invasiveness.

## Role of Adenosine Metabolizing Ecto-Enzymes and Signals in Glioblastoma Aggressiveness

CD73 and Prostatic Acid phosphatase (PAP), which convert AMP to ADO, play a role in GBM progression. In particular, CD73 is expressed in GSCs from primary human tumors and glioma cell lines (D’Alimonte et al., 2015; Azambuja et al., 2019) and its overexpression is an important feature for glioma cell adhesion and tumor cell-extracellular matrix interactions (Cappellari et al., 2012). Accordingly, CD73 downregulation decreases GBM growth in *vitro/in vivo* models (Azambuja et al., 2019) and is coupled to a better outcome of GBM patients (Xu et al., 2013). Further data obtained in GSCs showed that AMP degradation by PAP is prevalent in hypoxic condition (Torres et al., 2019), increasing extracellular ADO levels (30–200 nM) up to 100 times (Uribe et al., 2017). As well, ADO levels measured in the extracellular fluid of glioma tissue from patients are elevated, being in the low micromolar range (Melani et al., 2003). Interestingly, increased ADO levels promoted tumorigenic GSC characteristics (Quezada et al., 2013; Garrido et al., 2014; Torres et al., 2016), while significantly increasing cell proliferation. In contrast, extracellular AMP, when present at high concentrations (1–3 mM), decreased proliferation of U138MG glioma cells lines (Bavaresco et al., 2008).

As for receptors involved in pro-/anti-tumorigenic ADO effects, all four receptor subtypes (namely, A1, A2A, A2B, and A3) are expressed in GSCs (D’Alimonte et al., 2015). The involvement of ADO/related analogues in the growth/recurrence of gliomas gave rise to conflicting results about the role played by A1 and/or A2 receptors (see Gessi et al., 2011; Ceruti and Abbracchio, 2020). For example, some findings showed that A1 and A2B receptor stimulation had a prominent anti-proliferative/pro-apoptotic effect on GSCs *via* distinct regulation of the kinetics of ERK/AKT phosphorylation and the expression of hypoxia-inducible factors. Moreover, ADO receptor agonists sensitized GSCs to temozolomide (TMZ) toxicity and prolonged its effects (Daniele et al., 2014). In contrast, Yan et al. (2019) recently demonstrated that A2B receptor expression showed a 20-fold increase in GBM cells implanted in mice brain and their blockade potently increased GBM cell death induced by TMZ by downregulating multidrug resistance transporter function. Likely, the differences above reported may depend on different experimental models used in the two studies. Noteworthy, both A2A and A2B receptors may be involved in the regulation of angiogenesis, indirectly supporting tumor growth with oxygen and nutrients (Gessi et al., 2011). In contrast, there is a more general agreement on the role of the low affinity ADO/A3 receptors (A3R) on GBM growth/recurrence. Indeed, GSC migration and invasion was promoted by activation of these receptors especially in hypoxic condition (Torres et al., 2019). Accordingly, the blockade of CD73 together with that of ADO receptors decreased the adhesion of cultured GBM cell lines (U138MG) to the extracellular matrix. A3R stimulation also promoted the expression of EMT markers in GSCs obtained from primary tumors and human U87MG cell line.

## Role of Intracellular Purine Alterations in Glioblastoma Malignancy

In addition to alterations of membrane/extracellular purinome components, modifications of intracellular purine metabolism may also be relevant in GBM. For instance, the presence of high extracellular ADO levels usually activates its uptake by selective carriers, mainly equilibrative transporters (ENTs), whose driving force is the difference in nucleoside concentration across cell membrane. Thus, in rat C6 glioma cells at least, ADO uptake by these carriers, in particular ENT2 subtype, led to intracellular AMP accumulation due to activity of cytosolic ADO kinase functioning, however, only in oxygenation condition. AMP elevation, in turn, produced cell growth inhibition through pyrimidine starvation (Ohkubo et al., 2007). Other findings concern enzymes metabolizing other purines. Thus, in GBM ADF cell line, hyperactivity of the ubiquitous cytosolic enzyme 5’-nucleotidase II, converting inosines/guanosine monophosphate into the respective nucleosides, was accompanied by increased cell proliferation/resistance to gemcitabine and mitomycin C (Cividini et al., 2015). Finally, in GBM it was frequently found homozygous deletion of methylthioadenosine phosphorylase (MTAP), an enzyme working in the purine/methionine salvage pathway and metabolizing methylthioadenosine generated during polyamine biosynthesis to eventually produce adenine and methionine. MTAP alterations favor GSC formation with increased expression of the aggressiveness marker CD133 (Hansen et al., 2019), which is associated with poor clinical outcome of GBM patients (Zeppernick et al., 2008). Interestingly, CD133^+^ GSCs were killed by inhibiting *de novo* purine synthesis with l-alanosine, a potent low toxicity inhibitor of adenine biosynthesis, as reported in other tumors (Lubin and Lubin, 2009), coupled to ADO reuptake inhibition.

## Discussion

Being impossible to summarize the huge amount of data on GBM-purines relationship, we herein collected some impressive purinome dysfunctions in GBM. As for extracellular components, in our opinion two of them are crucial in supporting an oncogenic role of ATP/ADO in GBM. One is the activity of enzymes deputed to ATP or AMP metabolism, which is decreased or increased, respectively. This imbalance, coupled to enhanced purine loss from hypoxic/dying tumor cells, raises the extracellular ATP/ADO levels. The other one is a major expression of low affinity purine receptors, such as P2X7R or A3R, in GSCs and/or GBM cell lines, at least (Figure 1). The simultaneous presence of these alterations on the same tumor cell allows ATP/ADO to interact with receptors normally activated only by increased levels of the two purines, causing a more prolonged survival and/or aggressive behavior of the tumor itself.

While the role/modulation of CD39/CD73 activity in GBM still needs further elucidation in *vivo* experimental models/patients and the extracellular purine level measurement is difficult, especially *in vivo*, given the rapidity by which these compounds are metabolized (nucleotides) or transported inside cells (nucleosides), at present search for A3R/P2X7R expression level in GBM surgical specimens seems more feasible, likely opening a new prognostic/therapeutic scenario. Thus, in the presence of ascertained receptor overexpression, it could be useful to use selective A3R/P2X7R antagonists to curtail support to tumor growth/malignancy. Noteworthy, the use of P2X7R antagonists might prove convenient also in inhibiting the onset of seizures occurring during GBM progression in patients, since ATP/P2X7R interaction increases the release of ATP, triggering a vicious cycle, and also of glutamate (Strong et al., 2018), which may be toxic to the surrounding healthy brain neurons/tissue, also accounting for seizures (Corbetta et al., 2019). Finally, a high P2X7R expression is a good prognostic factor for glioma radiosensitivity and survival probability in humans (Gehring et al., 2015).

As well, alterations in GBM intracellular metabolism of purines/related substances can be important, in light of recent data showing that purine synthesis is fundamental for GSC maintenance (Wang et al., 2017). In our opinion, GBM MTAP deficiency is appealing. However, since tumor markers in addition to CD133 have been identified in GSCs (Lathia et al., 2015), studies on purine metabolism alterations should be carried out also in other GSC types and related intracranial xenograft animal models to assess the existence of MTAP loss or further/different purine metabolism dysregulation in them. Afterward, it could be the case of evaluating the validity of modulating *de novo* purine synthesis/reuptake by *ad hoc* drugs.

In conclusion, the information collected here supports the idea that purinome alterations may have important consequences in events related to GBM aggressiveness/recurrence. Therefore, it is extremely important/needed to confirm whether these anomalies are present *in vivo*, so that they could be considered as new molecular markers of GBM and, hopefully, future targets for pharmacological/gene therapy.

## Glossary

ADO: adenosine
AK: adenosine kinase
A3R, A3: receptor
ATP,: adenosine tri
ADP,: adenosine di
AMP,: adenosine mono-phosphate
BzATP,: 2′(3′)-O-(4-benzoylbenzoyl)-ATP
EMT: epithelial-to-mesenchymal transition
E-NPPases: ectonucleoside pyrophosphatase/phosphodiesterases
ecto-5ʹ-NT/CD73: ecto-5ʹ-nucleotidase/CD73
E-NTPDases: ectonucleoside triphosphate diphosphohydrolase
ENTs: equilibrative transporters
GBM: glioblastoma multiforme
GSCs: glioblastoma stem cells
MTA: methylthioadenosine
MTAP: methylthioadenosine phosphorylase
NTAP: tissue-nonspecific alkaline phosphatase
NTs: nucleotidases
PAP: prostatic acid phosphatase
P2X7R, P2X7: receptor
SAH: S-adenosyl hydroxylase
